# International validation of the delirium prediction model for ICU patients (PREDELIRIC): a multicenter observational study

**DOI:** 10.1186/cc12336

**Published:** 2013-03-19

**Authors:** M van den Boogaard, L Schoonhoven, E Maseda, C Plowright, C Jones, A Luetz, PV Sackey, P Jorens, LM Aitken, F van Haren, JG van der Hoeven, P Pickkers

**Affiliations:** 1 Radboud University Nijmegen Medical Centre, Nijmegen, the Netherlands; 2 Radboud University Nijmegen Medical Centre, Scientific Institute for Quality of Healthcare, Nijmegen, the Netherlands; 3 Hospital Universitario La Paz, Madrid, Spain; 4 Medway Maritime Hospital, Kent, UK; 5 Whiston Hospital, Prescot, UK; 6 Charité - Universitaetsmedizin Berlin, Germany; 7 Karolinska University Hospital, Stockholm, Sweden; 8 University of Antwerpen, Belgium; 9 Princess Alexandra Hospital, Woolloongabba, Australia; 10 Canberra Hospital, Canberra, Australia

## Introduction

Delirium is a serious and frequent disorder in ICU patients. The aim of this study was to internationally validate the existing Prediction of Delirium ICU (PREDELIRIC) model to predict delirium in ICU patients.

## Methods

A prospective multicenter cohort study was performed in eight ICUs in six countries (Table 1). The 10 predictors (age, APACHE II score, urgent and admission category, infection, coma, sedation, morphine use, urea level, metabolic acidosis) were collected within 24 hours after ICU admission. CAM-ICU was used to identify ICU delirium.

**Table 1:** PREDELIRIC AUROC of different centers

	Delirium (%)	AUROC	95% CI
Belgium (%)	86 (15.2)	0.79	0.74 to 0.84
Germany (%)	60 (26.9)	0.85	0.80 to 0.91
Spain (%)	23 (18.0)	0.88	0.81 to 0.94
Sweden (%)	30 (39.0)	0.71	0.60 to 0.83
Australia Brisbane (%)	42 (12.8)	0.81	0.75 to 0.88
Australia Canberra (%)	23 (11.8)	0.80	0.72 to 0.89
UK_Prescot (%)	73 (30.8)	0.65	0.57 to 0.73
UK_Kent (%)	26 (36.6)	0.76	0.65 to 0.88
**PREDELIRIC overall **	**363 (19.9)**	**0.77**	**0.74 to 0.79**

## Results

A total of 2,852 adult ICU patients were screened and 1,824 (64%) were included. Main exclusion reasons were length of stay <1 day (19.1%) and sustained coma (4.1%). Mean ± CAM-ICU compliance was 82 ± 16% and inter-rater reliability 0.87 ± 0.17. Overall delirium incidence was 19.9% (range 27.2%). Despite significant differences between centres on all 10 predictors, the overall area under the receiver operating characteristic curve (AUROC) of the eight centers was 0.77 (95% CI: 0.74 to 0.79).

## Conclusion

The overall predictive value of the PREDELIRIC model was good, indicating that the prediction model can now be used internationally.

